# Genetic Diversity in the *SIR* Model of Pathogen Evolution

**DOI:** 10.1371/journal.pone.0004876

**Published:** 2009-03-16

**Authors:** Isabel Gordo, M. Gabriela M. Gomes, Daniel G. Reis, Paulo R. A. Campos

**Affiliations:** 1 Instituto Gulbenkian de Ciência, Oeiras, Portugal; 2 Centro de Matemática e Aplicações Fundamentais, Universidade de Lisboa, Lisboa, Portugal; 3 Departamento de Física, Universidade Federal Rural de Pernambuco, Recife-PE, Brazil; American Museum of Natural History, United States of America

## Abstract

We introduce a model for assessing the levels and patterns of genetic diversity in pathogen populations, whose epidemiology follows a susceptible-infected-recovered model (*SIR*). We model the population of pathogens as a metapopulation composed of subpopulations (infected hosts), where pathogens replicate and mutate. Hosts transmit pathogens to uninfected hosts. We show that the level of pathogen variation is well predicted by analytical expressions, such that pathogen neutral molecular variation is bounded by the level of infection and increases with the duration of infection. We then introduce selection in the model and study the invasion probability of a new pathogenic strain whose fitness (*R*_0_(1+*s*)) is higher than the fitness of the resident strain (*R*_0_). We show that this invasion probability is given by the relative increment in *R*_0_ of the new pathogen (*s*). By analyzing the patterns of genetic diversity in this framework, we identify the molecular signatures during the replacement and compare these with those observed in sequences of influenza A.

## Introduction

Understanding molecular variation in populations with a complex demographic history is of utmost importance [1], [2]. This is so, not only because most natural populations do not have simple demographic histories [3], but also because populations as those of microbes that can cause human diseases do not conform to a simple unstructured, constant size population model [4], [5]. In fact, the standard neutral model of M. Kimura [6], that has provided us with a null model against which we can create interesting alternative hypothesis to understand molecular evolution and variation, is far too simple for understanding pathogen genetic diversity. With this motivation we have studied a non-standard neutral model that aims at being simple enough, but not too simple, so as to account for some of the demographic processes that are likely to occur in natural pathogen populations. The susceptible-infected-recovered (*SIR*) framework has been used extensively in mathematical epidemiology [7], where the focus lies on how the prevalence and dynamics of infection varies with the transmission capacity of the pathogen and the characteristics of host immune response [8]. In this sense, pathogens are static entities whose evolution is disregarded, at least in the short term. But for pathogens with high mutation rates [9], such as RNA viruses, or even bacteria, it may not be safe to ignore pathogen evolution, even in the short term [10].

**Figure 1:**
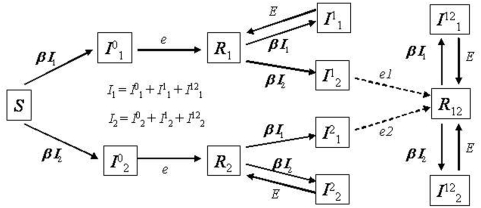
*SIR* model with reinfection and selection. *I^i^_,j_* means a host that has been infected with the *i* ^th^ strain and is currently carrying pathogen strain *j*. *e*_i_ are the different possible rates of recovery from the current infection, when all *e*_i_ are equal we have a neutral model with reinfection. R*_i_* means that the host has recovered from infection with strain *i*. We have simulated a strong selective advantage of the new strain by setting *e* = 0.1 and *E* = 7*e; *e*_1_ = 3*e and *e*_2_ = 7*e.

Recently, some epidemiological models have been studied where pathogen mutation has been incorporated [4], [5], [11], [12]. For example, Boni [11] studied an *SIR* model where pathogen mutation was introduced in a simple way. The model keeps track of various pathogen lineages that give rise to new lineages through mutation that implicitly occurs at the transmission stage. Furthermore, the population size of hosts is effectively infinite and intra-host drift is not considered. They show that if all new strains that are continuously created are selectively equivalent then diversity increases at a constant rate (*U*) and the number of extant lineages at a given time *t*, is Poisson distributed with mean *Ut*. In this model, the evolution within each host is not explicitly considered and *U* represents the rate of fixation of new mutations within a host. Other authors have used the powerful tool of coalescent theory [13] to analyse pathogen genetic diversity but have assumed that the pathogen populations follow the Wright-Fisher model of an unstructured population that fluctuates in size [14]. Here we introduce a modelling framework (Figure 1) that explicitly considers both the population structure of pathogens, which is related to the contact structure of their hosts, and intra-host evolution, where pathogens mutate and new strains can stochastically go extinct. Initially, we consider a neutral evolutionary process where every new strain, although genetically different, is phenotypically equivalent to any other strain, i.e. each strain has the same transmissibility and causes infections with the same duration. We ask what level and pattern of sequence diversity should be expected under this scenario, when both epidemiological and genetic equilibrium between mutation and drift are achieved. We then study the pattern of diversity when a new epidemic occurs. Finally, we introduce selection in the framework and compare the patterns of diversity expected in our model with those observed in natural influenza A isolates.

## Methods

### The *SIR* epidemiological model

In standard formulations of disease dynamics, the time evolution of the different classes of hosts is described by a simple set of ordinary differential equations[7]. Upon this assumption, the population is assumed to be homogeneous and infinitely large such that stochastic events are negligible. In the *SIR* model, the hosts can be in one of three states: susceptible (*S*), infected (*I*) and recovered (*R*). A susceptible host can get infected at rate *β* when in contact with infected individuals. At rate *τ* an infected individual will be recovered. Upon this dynamics the *SIR* model is then described by the following set of differential equations:
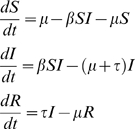
where *μ* corresponds to the birth and death rates of hosts. By measuring time in units of duration of infection, 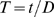, where 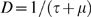, and considering the normalization condition, 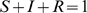, which implies that we can omit one of the equations, the model can be re-written as
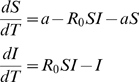
(1)where 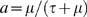, and 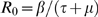 is the relevant parameter of the model and it is known as the basic reproductive number [7]. The basic reproductive number is the average number of secondary cases a typical infected individual can cause in a completely susceptible population during its entire infectious period. System (1) has two solutions: 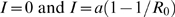. The disease-free equilibrium, 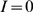, exists for every 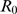 and is stable for 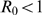, whereas the endemic equilibrium, 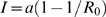, exists and is stable only when 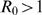.

Here, we study a discrete time *SIR* model where we consider explicitly the evolution of pathogens in a finite structured host population and where their genetic diversity is followed.

### Including pathogen genetic diversity in the *SIR* model

To study genetic diversity of a pathogen whose epidemiology follows the *SIR* model we consider a discrete model of a structured population [5], [15]. The population structure of pathogens is modeled as a metapopulation where each host is depicted as a deme in the metapopulation. *D* hosts are assumed. An empty deme represents a host in the susceptible state or in the recovered state, whereas a deme which is full corresponds to an infected host. A deme that is currently full (infected) can move to the recovered state with probability *e* (Figure 1). With probability *b* any given host can move to the susceptible state. So, the probability that a currently filled deme becomes empty is *e*+*b*. A deme that is currently empty and in the susceptible state can become full (infected) through transmission of pathogens- migrants- from nearby filled demes. This implies that the transmission rate, *β*, is proportional to the migration/recolonization rate, *m*. If an infection event occurs at a given time then, in the next time step, the pathogen reproduces with mutation (with mutation rate per genome per generation *U*) to give rise to a diverse population of size *N_d_*, which is the maximum level of parasites within an infected host.

We have considered a homogeneous contact structure, where any given host is connected to the remaining 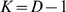 hosts. When a susceptible host is in contact with an infected host it can receive a given number of migrant pathogens which is assumed to be Poisson distributed with mean *N_d_m*. This implies that the mean level of transmission, *β*, corresponds to *N_d_mK*. An empty deme that is in the recovered state is not allowed to receive pathogens, which corresponds to the *SIR* model with no reinfection. As in the deterministic description of the *SIR* the relevant parameter is 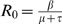, and considering small values of *e*, *b* and *N_d_m*, for which probabilities are similar to rates [16], in our time-discrete stochastic analogue the relevant parameter becomes:
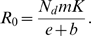
(2)


Therefore, we can estimate the proportion of infected hosts as:
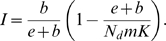
(3)


**Figure 2:**
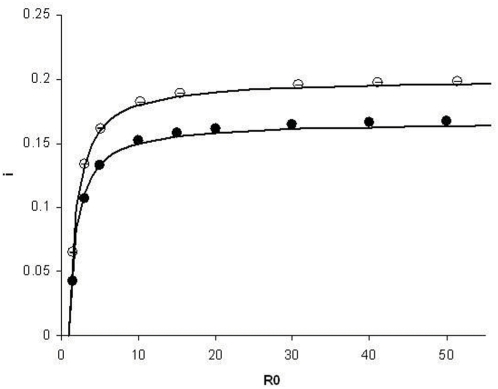
Fraction of infected individuals, I, as a function of the basic reproductive number *R*_0_. The parameter values are: *N_d_* = 10, *D* = 2000, *e* = 0.1 and *b* = 0.02 (full symbols) and *N_d_* = 20, *D* = 7000, *e* = 0.04 and *b* = 0.01 (empty symbols). The dashed-line is the theoretical prediction according to Equation (3).

We have compared the infection levels in the simulations with this expectation and found a clear agreement between Eq. (3) and our simulation results, which demonstrates the correspondence between the stochastic model with the fully connected host contact structure and the traditional deterministic *SIR* model (see Figure 2). We have checked that the results are not dependent on the total pathogen effective population size within hosts, *N_d_*, and on the number of demes *D*. We have also ascertained that 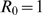 is the critical value to have a non-null probability of an outbreak occurrence.

The measures of genetic diversity that we have studied were the mean number of pairwise differences between sequences in random samples of the whole pathogen population (π) and the number of segregating sites. From these we calculated the statistic Tajima's D, whose expected value is zero under a constant size population following the Wright-Fisher model of neutral evolution. Genetic diversity was evaluated at equilibrium and also before epidemiological and genetic equilibrium was reached.

### Model for studying selection

Certain pathogens show rapid evolution [17], [18], [19], [20] and genetic analysis has strongly suggested the action of positive selection in some regions of their genomes. To understand the signature of selection on pathogen molecular variation in our model we started considering the invasion of the resident pathogen population by a slightly distinct variant (phenotypically). The new variant is assumed to have a higher fitness resulting from a lower rate of clearance by the infected host, but is it otherwise identical to the resident pathogen strain. In this way, a randomly chosen host is infected by the strain with higher *R*_0_, and the fate of this strain is followed in the population, until loss or fixation. By fixation, one means that the mutant has spread through the whole population and it is now not only the dominant strain but the only strain present. After simulating several thousands of independent simulation runs of this process, a fixation probability of the mutant is estimated as the number of independent simulations in which fixation occurred over the total number of simulations. The mean time to fixation is also obtained and it corresponds to the number of generations that the mutant takes since its appearance until its fixation.

### *SIR* model with reinfection and selection - a simple model for influenza A evolution

Influenza A is an RNA virus that causes annual winter epidemics in temperate climates, while circulating throughout the year in the tropics. With a high mutation rate, the population of influenza A virus can generate considerable genetic variability and if there would be no selection it could potentially attain high levels of genetic diversity. However, it has been found that its genetic diversity is reduced periodically (see [12]), and this is associated with cluster transitions. The evolutionary forces responsible for these patterns of molecular evolution are not well understood, although it is consensual that some form of selection is driving influenza A genome evolution [12], [21], [22], [23].

Previous modeling work has suggested that, in the case of influenza A, evolution occurs through the successive accumulation of neutral mutations, increasing viral diversity, followed by a sharp decline of the diversity which results from the fixation of a mutant strain that escaped host immune surveillance [12], [24]. Motivated by this, we introduce selection in our neutral model using a simple, yet insightful, way to understand influenza evolution. We assume that some level of reinfection can occur such that, while in the recovered state, a host can be reinfected with a probability, *β*. In this context we study a model where a new viral strain which is genetically sufficiently distinct (has accumulated a given number of mutations that where previously neutral), receives a selective advantage when it infects a host that had recovered from an initial infection caused by the old strain. The advantage is that the new strain causes a slightly longer infection in this host. Note though, that this new strain causes exactly the same duration of infection as the old one when it infects a host that has never been infected. A caricature of this model with all the relevant parameters can be seen in Figure 1. In this model we have studied the pattern of genetic diversity by introducing a genetic distance threshold, denoted by *d_c_*, by which pathogens carrying more than *d_c_* mutations, acquire a selective advantage.

### Sequence data of *Influenza* A virus

Complete coding sequences of the hemagglutinin (HA) gene of A/H3N2 influenza viruses from the New York state, USA, were collected from the NCBI Influenza Database [25] A file with the sequences is provided as a supplement (Sequences.fas Text S1). We calculate the genetic diversity and Tajima's D of 683 sequences from years 1993 to 2006 using DnaSP 4.20.2 [26]]. The analysis was made by seasons, in which a season was defined as the time-window between September and May.

## Results

### Level of genetic diversity

**Figure 3:**
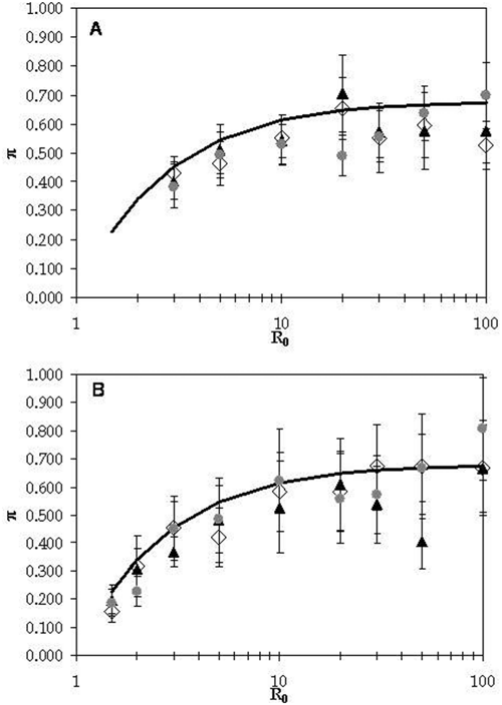
The level of genetic diversity in the pathogen population as a function of *R*_0_. Transmission is governed by the *SIR* model (1). Parameter values are as follows *D* = 30000, *e* = 0.1 *U* = 0.00005 and *b* = 0.005 (in panel A) and *U* = 0.00002 and *b* = 0.015 (in panel B), *N_d_* = 5 for filled triangles, *N_d_* = 10 for empty diamonds and *N_d_* = 20 for grey circles. The solid black line is the expected level of diversity as given by equation (4). In panel A for the lowest values of *R*_0_ the pathogen population could not be maintained.

We have studied the levels and patterns of genetic diversity in a pathogen population whose dynamics follows the *SIR* epidemiological model. Figure 3 displays the level of diversity observed in random samples from the entire pathogen metapopulation as a function of *R*_0_. In the figure, the increase of the parameter *R*_0_ was performed by the incrementing the migration rate *m*, i.e., by increasing the rate of transmission, while keeping all other parameters fixed. Intuitively one can expect that the level of diversity increases with the level of infection, since the overall population size increases; we also expect the level of diversity to increase with the pathogen mutation rate. Furthermore it has been shown [27] that in a metapopulation with extinction and recolonization genetic diversity decreases with increasing extinction rates. More formally we find that the level of diversity π is well approximated by the simplest expression
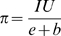
(4)where I is given by equation (3), which shows that neutral genetic diversity is proportional to the level and duration of infection and the mutation rate.

We also note that equation (4) performs best when host population turnover is much slower than recovery from infection (i.e. when *b* is much lower than *e*). From Figure 3 and several other simulations that we have performed, we find that this theoretical curve provides a better fit to the simulated genetic diversity when *b/e* is small, with deviations appearing when both this ratio and *R*_0_ are large (compare panel A in which *b/e* = 0.05 and panel B for which *b/e* = 0.15).

Many common infectious diseases, such as influenza where we focus later on, have low *R*_0_ (around 2–4) [28] and very low ratios *b/e* (around 0.0002), ensuring the applicability of equation (4) as a good approximation to the level of genetic diversity that should generally be expected under neutral evolution of many infectious diseases.

### Frequency spectrum of neutral mutations

We also studied how the frequency of segregating neutral mutations in this metapopulation model compares with that expected under the standard neutral model of molecular evolution. In order to do so we have measured the average Tajima's D statistic in samples of the simulated pathogen population. We always found values of Tajima's D similar to that expected for the standard neutral model, i.e, average values of D∼0 (see Table 1 for some examples). This implies that demographic structure, such as that studied here, is difficult to detect with a classical measure of deviations from the standard neutral frequency spectrum.

**Table 1:** Frequency distribution of mutations assessed by measuring Tajima's D.

*b* = 0.005				*b* = 0.015			
*N_d_*	*R*_0_	*D*	2SE	*N_d_*	*R*_0_	*D*	2SE
5	3	0.06	0.02	5	1.5	0.01	0.08
5	5	0.09	−0.02	5	2	0.04	0.14
5	10	0.09	−0.05	5	3	−0.11	0.12
5	20	0.13	0.03	5	5	−0.08	0.18
5	30	0.09	−0.10	5	10	−0.06	0.20
5	50	0.13	0.04	5	20	−0.04	0.19
5	100	0.11	−0.05	5	30	−0.04	0.19
20	3	−0.08	0.12	5	50	−0.15	0.16
20	5	−0.05	0.10	5	100	0.04	0.17
20	10	−0.09	0.10	10	1.5	−0.08	0.07
20	20	−0.14	0.10	10	2	0.02	0.14
20	30	−0.14	0.11	10	3	0.04	0.14
20	50	−0.09	0.12	10	5	−0.21	0.13
20	100	−0.05	0.14	10	10	−0.06	0.16

Fixed parameters were *D* = 10000, *U* = 0.0001 and *e* = 0.1.

### Approach to Equilibrium

**Figure 4:**
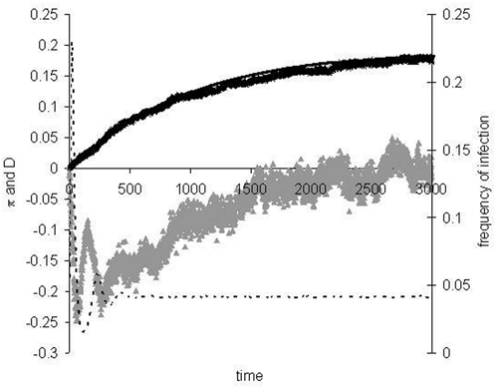
Approach to equilibrium *R*_0_ = 2.5. The parameter values are *D* = 5000, *U* = 0.0001, *e* = 0.1, *b* = 0.008 and *N_d_* = 10. Dashed line is the level of infection in the population, crosses is the average pairwise diversity as a function of time (in generations), the black line is the prediction of equation (5), using the level of infection obtained in the simulations. Grey triangles represent the mean value of Tajima's D statistic. In the initial period of the epidemic, diversity increases with slope of approximately 2*U* (linear regression slope 0.0002, *R* ^2^ = 0.99).

In many situations, we may be interested in knowing the level of diversity before equilibrium is attained. This is particularly relevant in the period during or after an epidemic [11]. We have studied this by simulation (Figure 4) and considered a heuristic approximation for the average level of diversity as follows. Let us suppose that the whole convergence to equilibrium can be approximated by a change in pathogen population size which starts from 1 individual. Initially there is no diversity and when 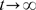 the level of diversity will be approximately 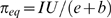 where 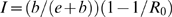 with 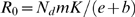, as we have seen before.

Tajima [29] has shown that for a single unstructured population that fluctuates in size (*N_t_*), the number of segregating sites, *S*, in a sample of size *n*, changes in time according to
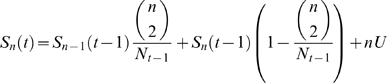
which for 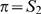 where 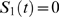 gives
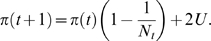



Because at equilibrium the level and pattern of genetic diversity is similar to that expected under a standard neutral model with effective population size of 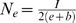 we try the following heuristic approximation for the variation in diversity levels with time:
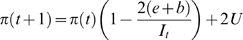
(5)where *I_t_* is approximated by the number of infected hosts in the *SIR* model.

Figure 4 shows that equation (5) provides a good approximation for the level of diversity as the population approaches both epidemiological and genetic equilibrium. We can observe that at the peak of the epidemic, diversity levels are low and Tajima's D is very negative. After the initial epidemic, both diversity levels and Tajima's D start to increase. Diversity initially increases at a rate 2*U* approximately and follows very closely the levels predicted by equation (5).

### Invasion of new pathogenic strains

**Figure 5:**
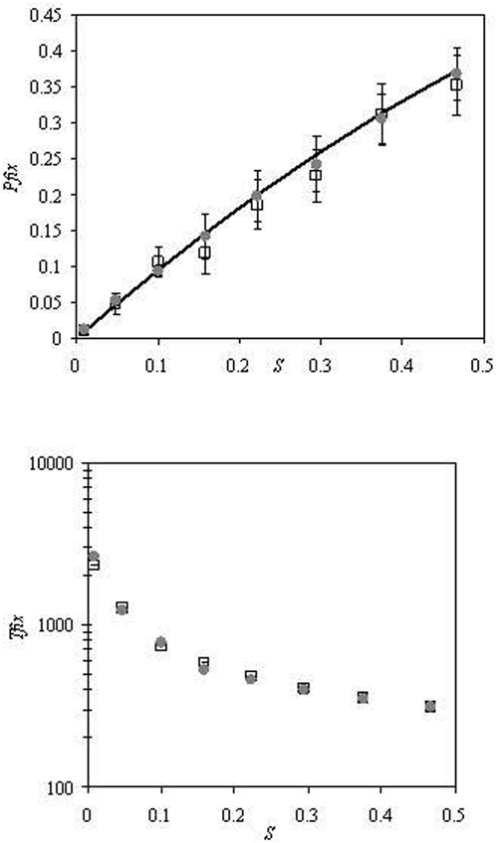
Probability and mean time to replacement. *D* = 5000, *b* = 0.01, *e* = 0.1 and initial *R*_0_ = 4.5. There is no reinfection. *N_d_* = 10 (circles) and *N_d_* = 20 (squares) A) Probability of replacement of new strain that has selective advantage s (*P_fix_*). B) Mean time to replacement of new selected strain (*T_fix_*).

We have considered the case where a new strain that has a higher *R*_0_ is introduced in the population. This new strain is assumed to carry some beneficial mutation that makes it more virulent in the sense that is causes a longer infection in the host. We then asked what the probability of such virulent strain to invade is and, on average, how long this invasion takes. As commonly done in the population genetics literature [30], we define the relative selective advantage of the new strain as 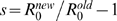. We show in Figure 5 the simulation results of several independent introductions of mutant strains with different selective advantages. We can see that the probability of replacement depends on the selective advantage (*s*) according to 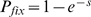. Since in this model, to a very good approximation, any given host will either be infected with the old strain or with the new strain, and the number of infected hosts is approximately constant, the process of fixation can be well approximated by a simple Moran model of birth and death of infected hosts, where the probability of birth of infected hosts carrying the new strain is slightly higher than that of infected hosts carrying the old strain [31]. Under the Moran model the probability of fixation of a beneficial mutant is 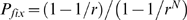 where *r* is the fitness of the new mutation and *N* is the population size. In our case *r* = 1+*s* and *N* corresponds to the number of infected hosts, which leads to the probability of fixation given above.

### Pattern of diversity under the invasion of escape mutant strains

We now study a model where we have introduced selection in a simple, yet relevant, way for understanding influenza evolution. We have assumed that, after recovery from a first infection, hosts can be reinfected (see Figure 1). If a host experiences a second infection with a strain that is genetically distant from the one that caused the first infection, then its clearance rate is lower than if the infection would have been caused by a genetically similar virus (*e*_1_<*E* and *e*_1_<*e*_2_). This simply says that the repertoire of antibodies that was built upon infection with a given pathogen will not be optimal against an antigenically distinct pathogen. Moreover, we assume that this effect is asymmetric. An infection with the invading strain that is preceded by an infection with resident strain has a clearance rate that is lower than if the order is reversed (*e*_1_<*e*_2_). The argument is that the invading strain emerged in the presence of antibodies against the resident and escaped successfully, while the reverse is not true. The resident emerged before the maturation of antibodies against the invader, and therefore has never been under their selective pressure.

**Figure 6:**
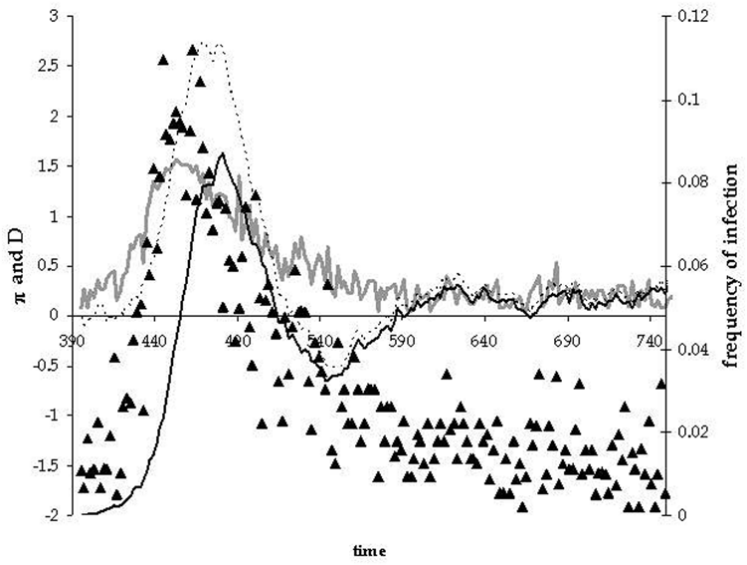
Time plot of the pattern of diversity during the replacement of a new strain. On the left scale, we plot π (gray line) and Tajima's D (filled triangles). On the right scale, we plot the total frequency of infection (dashed line) and the frequency of hosts infected with new selected strain (filled line). Parameters are as follows: *D* = 30000, initial *R*_0_ = 4, *U* = 0.0001, *d_c_* = 2 *e* = 0.1, E = 0.7, *e*_1_ = 0.3, *e*_2_ = 0.7 and *b* = 0.005.

**Figure 7:**
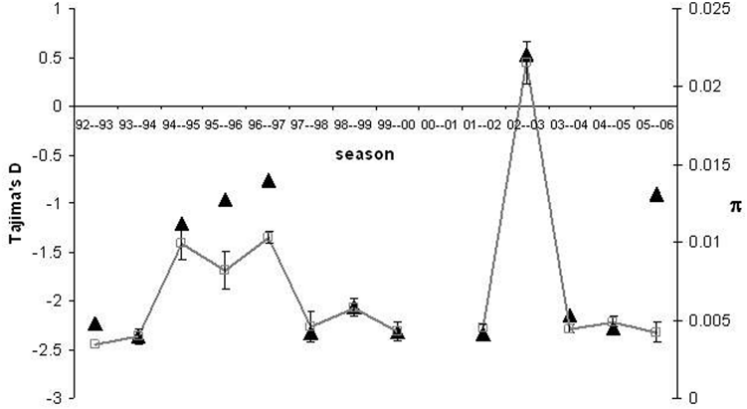
Pattern and level of genetic diversity in the coding region of hemagglutinin gene of A/H3N2 influenza virus. Sequences sampled in New York State, USA, over several seasons, in which a season was defined as the time-window between September and May. Across the time period analysed Tajima's D (full triangles) is negative but in 2002–2003 we can observe that Tajima's D achieves a positive value which accompanies an increase in genetic diversity π (represented as open squares).

To follow the level and pattern of genetic diversity in the sequences we have assumed that two strains are antigenically distinct when they differ by two or more mutations. Figure 6 illustrates what we have observed over many different simulations. As the new strain invades the population it leaves a molecular signature in genetic sequences sampled randomly from the pathogen population. As can be seen in the figure (see also Supporting Information Figure S1, Figure S2 and Figure S3), as the new strain sweeps through the population, the average level of pairwise differences between sequences increases and this is accompanied by a substantial increase in the value of Tajima's D, which becomes positive. When the new strain becomes dominant in the population, diversity decreases to very low levels and this is accompanied by a change in sign of Tajima's D, which now becomes consistently negative. So a rapid and drastic change in sign of Tajima's D and a corresponding decrease in diversity is a molecular signature of a new strain becoming dominant in the population. In Figure 7 we have plotted the values of π and Tajima's D for the HA gene of influenza A sampled in New York. Interestingly, we can observe that in the seasons 2002–2003 and 2003–2004 a rapid increase in diversity accompanied by an increase in Tajima's D, which become positive, followed by a rapid decrease in diversity and a change in sign of Tajima's D.

In Table 2 we show the probability that the new strain replaces the old strain and also show on average how rapidly that replacement takes place. From the results in Table 2 it is clear that, under the described selection (*e*_1_<*e*_2_), the probability of fixation of the new strain is always higher than the probability of replacement by a neutral strain (*e*_1_ = *e*_2_) and that the time for it to sweep through the population can be short. As the mechanism of selection of the new strain over the resident acts upon reinfection with the asymmetry *e*_1_/*e*_2_<1, we can see that as this ratio becomes smaller the probability of replacement increases and the mean time to replacement decreases, all else being equal.

**Table 2:** Combinations of model parameters used in simulations.

*D*	R_0_	*e*	*E*	*e*1	*e*2	*b*	P_fix_	T_fix_
**30000**	4	0.1	0.7	0.1	0.7	0.005	0.65 (0.06)	301
	4	0.1	0.7	0.2	0.7	0.005	0.53 (0.06)	621
	4	0.1	0.7	0.3	0.7	0.005	0.41 (0.06)	1042
	4	0.1	0.7	0.5	0.7	0.005	0.11 (0.06)	2358
	4	0.1	0.7	0.6	0.7	0.005	0.05 (0.04)	5184
	4	0.1	0.7	0.1	0.6	0.005	0.67 (0.09)	1809
	3.2	0.1	0.7	0.6	0.7	0.005	0.03 (0.03)	6792
	3.2	0.1	0.7	0.5	0.7	0.005	0.04 (0.04)	2753
	3.2	0.1	0.7	0.3	0.7	0.005	0.22 (0.05)	1047
	3.2	0.1	0.7	0.2	0.7	0.005	0.48 (0.06)	597
	3.2	0.1	0.7	0.1	0.7	0.005	0.60 (0.06)	250
**10000**	4	0.1	0.7	0.1	0.35	0.005	0.34 (0.09)	771
	4	0.1	0.7	0.1	0.5	0.005	0.66 (0.09)	383
	4	0.1	0.7	0.1	0.6	0.005	0.72 (0.09)	297
	4	0.1	0.7	0.1	0.7	0.005	0.71 (0.09)	150

Fixed parameters were *N_d_* = 5 and *U* = 0.0001.

## Discussion

We have developed a simulation framework aiming at establishing the expectations for levels and patterns of neutral genetic diversity under the epidemiological *SIR* model. Unlike previous modeling frameworks [11], [12], [23], we have introduced a demographic structure where both within- and between-host evolutionary processes can be studied. This framework should be applicable to many different pathogens. We have found that DNA/RNA sequence variability is not only proportional to the level of infection in the population but also depends specifically on the duration of individual infection, such that, for the same prevalence of infection, pathogens which cause longer infections can sustain more genetic variability. For example, the genetic diversity is 0.26±0.04 for *e* = 0.2, but for a longer infection period (*e* = 0.1) the genetic diversity is 1.11±0.20 (when R_0_ = 20, *D* = 30000, *N_d_* = 10, U = 0.00001 and *b* = 0.01). When extending this model to incorporate selection amongst strains that are continuously generated through mutation, we found that simple forms of selection will lead to simple predictions for the probability of replacement of new strains. For concreteness, imagine that a new strain is introduced in a population, either through mutation of immigration. The only phenotypic change in this new strain is a small increase in the duration of infection, which increases significantly the chance of replacement. As an example, a 10% increase in the duration of infection leads to a 10 fold increase in the chance of replacement.

We then studied strain phenotypic diversity generated by mutation, which will lead to antigenic differences in a pathogen, such as the influenza A virus, and incorporated those in an *SIR* model with reinfection. Despite its simplicity, the model tries to capture genetic properties of influenza A drift evolution. Influenza A evolution is roughly characterized by two evolutionary phenomena: shift and drift [22]. Shift events are associated with subtype replacements and, typically, cause pandemics. Between shifts, antigenic drift occurs. This is characterized by the accumulation of mutations generating viral diversity. The majority of mutations are thought to be neutral but some, or certain combinations, can lead to an antigenically distinct virus which will be subject to selection since it has a reproductive advantage. Motivated by what has been suggested for the evolution of influenza A, we have followed the patterns of sequence diversity under a model where we assumed that, after the accumulation of a critical number of neutral mutations, the pathogenic strain would have a reproductive advantage. We found two clear molecular signatures of replacement in the model: a rapid reduction in diversity and a change in the sign of Tajima's *D* (from positive to negative), as replacement occurs. This molecular signature is observed in sequences from influenza A over the seasons between 2001 and 2004.

A debate has arisen concerning the possibility that influenza A drift evolution is driven by continuous positive Darwinian selection [21], or by epochal selection [12], [24]. Our model makes clear predictions on the molecular signatures of each scenario provided that data from sufficiently frequent sample exists. If continuous positive selection is occurring we should expect to see repeated molecular signatures of replacement (repeated decreases in π co-occurring with continuous changes in sign of Tajima's *D*), as in Figure 6. If long periods of neutral evolution occur then no such pattern is expected, as in Figure 4.

The model presented combines pathogen transmission, mutation and selection under minimal assumptions that are verified by many pathogens. This makes the results and conclusions widely applicable.

## Supporting Information

Figure S1 Time plot of the pattern of diversity during the replacement of a new strain. On the left scale of the plot diversity (gray line) and Tajima's D (filled triangles). On the right scale we plot the total frequency of infection (dashed line) and the frequency of hosts infected with new selected strain (filled line). Parameters are as follows: D = 30000, initial R0 = 4, U = 0.0001, dc = 2 e = 0.1, E = 0.7, e1 = 0.2, e2 = 0.7 and b = 0.005.
(0.04 MB TIF)

Figure S2 Time plot of the pattern of diversity during the replacement of a new strain. On the left scale of the plot diversity (gray line) and Tajima's D (filled triangles). On the right scale we plot the total frequency of infection (dashed line) and the frequency of hosts infected with new selected strain (filled line). Parameters are as follows: D = 10000, initial R0 = 4, U = 0.0001, dc = 2 e = 0.1, E = 0.7, e1 = 0.3, e2 = 0.7 and b = 0.005.
(0.04 MB TIF)

Figure S3 Time plot of the pattern of diversity during the replacement of a new strain. On the left scale of the plot diversity (gray line) and Tajima's D (filled triangles). On the right scale we plot the total frequency of infection (dashed line) and the frequency of hosts infected with new selected strain (filled line). Parameters are as follows: D = 30000, initial R0 = 4, U = 0.00005, dc = 2 e = 0.1, E = 0.7, e1 = 0.3, e2 = 0.7 and b = 0.005.
(0.04 MB TIF)

Text S1 Supporting sequence information.
(1.30 MB DOC)
