# Supplementary figures and images for: Genetic Diversity in the SIR Model of Pathogen Evolution
Source: PLoS One. 2009 Mar 16;4(3):e4876. doi: 10.1371/journal.pone.0004876 (PMC2653725; doi:10.1371/journal.pone.0004876)

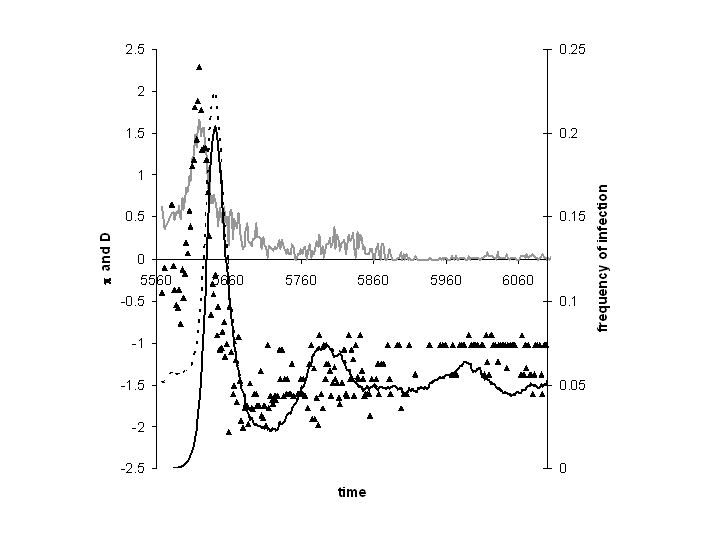

Supplement: Figure S1 — Time plot of the pattern of diversity during the replacement of a new strain. On the left scale of the plot diversity (gray line) and Tajima's D (filled triangles). On the right scale we plot the total frequency of infection (dashed line) and the frequency of hosts infected with new selected strain (filled line). Parameters are as follows: D = 30000, initial R0 = 4, U = 0.0001, dc = 2 e = 0.1, E = 0.7, e1 = 0.2, e2 = 0.7 and b = 0.005. (0.04 MB TIF) [file pone.0004876.s001.tif]

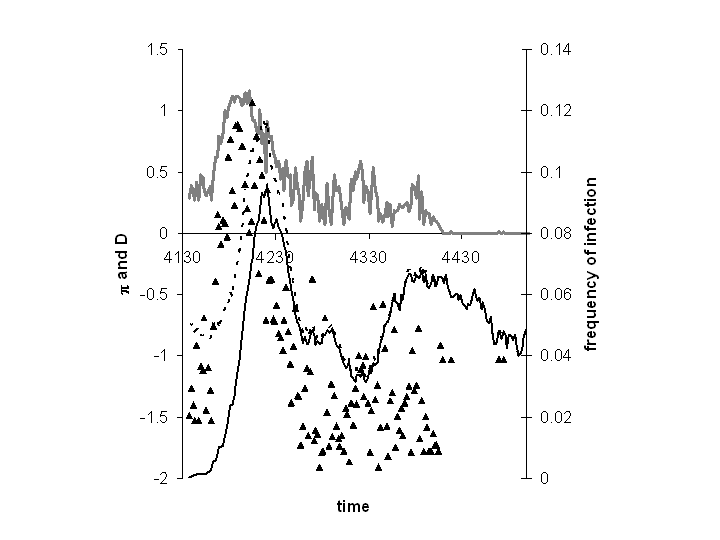

Supplement: Figure S2 — Time plot of the pattern of diversity during the replacement of a new strain. On the left scale of the plot diversity (gray line) and Tajima's D (filled triangles). On the right scale we plot the total frequency of infection (dashed line) and the frequency of hosts infected with new selected strain (filled line). Parameters are as follows: D = 10000, initial R0 = 4, U = 0.0001, dc = 2 e = 0.1, E = 0.7, e1 = 0.3, e2 = 0.7 and b = 0.005. (0.04 MB TIF) [file pone.0004876.s002.tif]

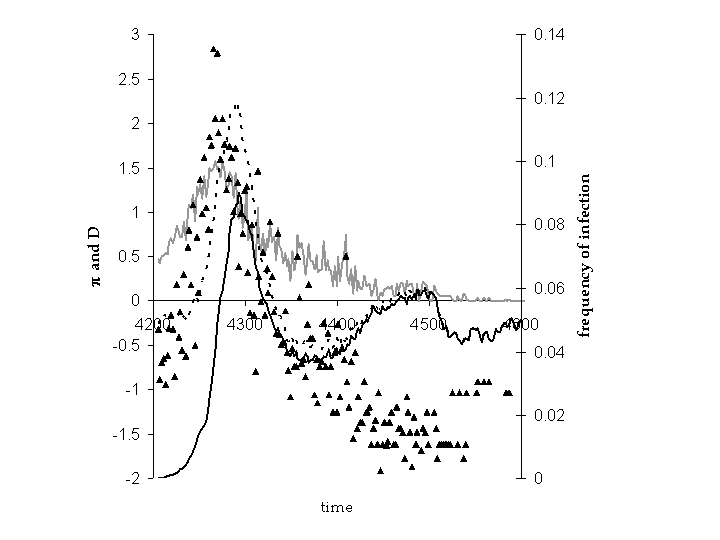

Supplement: Figure S3 — Time plot of the pattern of diversity during the replacement of a new strain. On the left scale of the plot diversity (gray line) and Tajima's D (filled triangles). On the right scale we plot the total frequency of infection (dashed line) and the frequency of hosts infected with new selected strain (filled line). Parameters are as follows: D = 30000, initial R0 = 4, U = 0.00005, dc = 2 e = 0.1, E = 0.7, e1 = 0.3, e2 = 0.7 and b = 0.005. (0.04 MB TIF) [file pone.0004876.s003.tif]
